# Simultaneous Estimation of Cinnamaldehyde and Eugenol in Essential Oils and Traditional and Ultrasound-Assisted Extracts of Different Species of Cinnamon Using a Sustainable/Green HPTLC Technique

**DOI:** 10.3390/molecules26072054

**Published:** 2021-04-03

**Authors:** Ahmed I. Foudah, Faiyaz Shakeel, Mohammed H. Alqarni, Samir A. Ross, Mohammad A. Salkini, Prawez Alam

**Affiliations:** 1 Department of Pharmacognosy, College of Pharmacy, Prince Sattam Bin Abdulaziz University, Al-Kharj 11942, Saudi Arabia; a.foudah@psau.edu.sa (A.I.F.); m.alqarni@psau.edu.sa (M.H.A.); m.salkini@psau.edu.sa (M.A.S.); 2 Department of Pharmaceutics, College of Pharmacy, King Saud University, Riyadh 11451, Saudi Arabia; faiyazs@fastmail.fm; 3 National Center for Natural Products Research, University of Mississippi, Oxford, MS 38677, USA; sross@olemiss.edu; 4 Department of Biomolecular Sciences, School of Pharmacy, University of Mississippi, Oxford, MS 38677, USA

**Keywords:** cinnamaldehyde, eugenol, plant extract, sustainable HPTLC, simultaneous estimation

## Abstract

A wide range of analytical techniques are reported for the determination of cinnamaldehyde (CCHO) and eugenol (EOH) in plant extracts and herbal formulations either alone or in combination. Nevertheless, sustainable/green analytical techniques for the estimation of CCHO and EOH either alone or in combination are scarce in the literature. Accordingly, the present research was carried out to establish a rapid, highly sensitive, and sustainable high-performance thin-layer chromatography (HPTLC) technique for the simultaneous estimation of CCHO and EOH in the traditional and ultrasound-assisted methanolic extracts of *Cinnamomum zeylanicum,* *C. burmannii*, and *C. cassia* and their essential oils. The simultaneous estimation of CCHO and EOH was performed through NP-18 silica gel 60 F254S HPTLC plates. The cyclohexane/ethyl acetate (90:10, *v v*^−1^) solvent system was optimized as the mobile phase for the simultaneous estimation of CCHO and EOH. The greenness score of the HPTLC technique was predicted using AGREE software. The entire analysis was carried out at a detection wavelength of 296 nm for CCHO and EOH. The sustainable HPTLC technique was observed as linear in the range 10–2000 ng band^−1^ for CCHO and EOH. The proposed technique was found to be highly sensitive, rapid, accurate, precise, and robust for the simultaneous estimation of CCHO and EOH. The content of CCHO in traditional methanolic extracts of *C. zeylanicum,* *C. burmannii*, and *C. cassia* was found to be 96.36, 118.49, and 114.18 mg g^−1^, respectively. However, the content of CCHO in ultrasound-assisted methanolic extracts of *C. zeylanicum,* *C. burmannii*, and *C. cassia* was found to be 111.57, 134.39, and 129.07 mg g^−1^, respectively. The content of CCHO in essential oils of *C. zeylanicum,* *C. burmannii*, and *C. cassia* was found to be 191.20, 214.24, and 202.09 mg g^−1^, respectively. The content of EOH in traditional methanolic extracts of *C. zeylanicum,* *C. burmannii*, and *C. cassia* was found to be 73.38, 165.41, and 109.10 mg g^−1^, respectively. However, the content of EOH in ultrasound-assisted methanolic extracts of *C. zeylanicum,* *C. burmannii*, and *C. cassia* was found to be 87.20, 218.09, and 121.85 mg g^−1^, respectively. The content of EOH in essential oils of *C. zeylanicum,* *C. burmannii*, and *C. cassia* was found to be 61.26, 79.21, and 69.02 mg g^−1^, respectively. The amounts of CCHO and EOH were found to be significantly higher in ultrasound-assisted extracts of all species compared to its traditional extraction and hence ultrasound extraction has been proposed as a superior technique for the extraction of CCHO and EOH. The AGREE analytical score of the present analytical technique was predicted as 0.75, suggesting excellent greenness profile of the proposed analytical technique. Based on all these observations and results, the proposed sustainable HPTLC technique can be successfully used for the simultaneous estimation of CCHO and EOH in different plant extracts and herbal products.

## 1. Introduction

Cinnamon has been used as an old spice for a long time [1]. Cinnamon has also shown various therapeutic effects and has characteristic flavor and fragrance [1,2]. Cinnamon is obtained from the dried inner bark of several species of *Cinnamomum*, which includes *Cinnamomum zeylanicum* Nees, *Cinnamomum burmannii* (Nees & T.Nees) Blume, *Cinnamomum cassia* (L.) J.Perl, and *Cinnamomum loureirii* Nees etc. [1]. Cinnamon belongs to the family Lauraceae [2]. Cinnamon is a rich source of essential oils and phenols [1,2]. The main phytoconstituents of different species of cinnamon bark and cinnamon oils are cinnamaldehyde (CCHO) and eugenol (EOH). Cinnamon has antioxidant [3], anti-diabetic [4], anti-spasmodic [5], carminative [6], antiseptic [7], and anti-microbial [7,8] qualities. 

Thorough literature analysis has suggested a wide range of analytical techniques for the determination of CCHO and EOH either alone or in combination with other phytoconstituents. An ultra-violet (UV) spectrometry technique was applied for the analysis of CCHO in its crude drugs and herbal preparations [9]. Different high-performance liquid chromatography (HPLC) techniques were also used for the analysis of CCHO either alone or in combination with other phytoconstituents in plant extracts and herbal preparations [10,11,12]. An HPLC technique was also used for the determination of CCHO in combination with cinnamic acid in rat plasma [13]. Some gas-chromatography mass-spectrometry (GC-MS) techniques have also been reported for the analysis of CCHO either alone or in combination with other natural compounds [14,15]. GC-MS technique has also been reported for the quantification of CCHO and its metabolites in rat tissues [16]. The gas-chromatography flame-ionization detector (GC-FID) technique was also used for the estimation of CCHO in combination with other natural compounds in commercial biopesticides [17]. A high-performance thin-layer chromatography (HPTLC) technique has also been reported for the estimation of CCHO in *C. zeylanicum* bark powder [18]. A UV spectrometry technique using liquid–liquid microextraction was applied for the analysis of EOH in personal care products [19]. Different HPLC methods were also used for the determination of EOH in different plant extracts and herbal preparations [19,20,21,22]. Various HPTLC techniques have also been reported for the determination of EOH in plant extracts and herbal preparations [23,24,25]. Some GC-MS techniques have also been reported for the determination of EOH in clove extracts [26,27]. Electrochemical and voltammetry techniques have also been reported for the determination of EOH in clove oil [28,29]. GC-FID technique has also been reported for the simultaneous estimation of CCHO and EOH along with other natural compounds in traditional Chinese medicinal preparations [30]. An HPLC method was also used for the simultaneous estimation of CCHO and EOH in the stem bark of *C. zeylanicum* [31]. Liquid chromatography mass-spectrometry (LC-MS) and HPLC techniques were also applied for the simultaneous estimation of CCHO and EOH in combination with other phytoconstituents in Chinese herbal medicine [32]. HPTLC technique was also applied for the simultaneous determination of CCHO and EOH in combination with other phytoconstituents in plant extracts [2,33]. Based on thorough literature analysis, it has been found that a wide range of analytical techniques was used for the quantification of CCHO and EOH either alone or in combination with other phytoconstituents. Unfortunately, the greenness profile of literature analytical techniques was not reported and determined. Recently, the analytical techniques related to green analytical chemistry (GAC) or environmentally benign analytical chemistry are enhancing day by day for the determination of natural/herbal compounds in their plant extracts and herbal formulations [34,35,36,37,38,39]. Various analytical/metric approaches have been used for the prediction of greenness profiles of the analytical techniques [40,41,42,43,44]. Among them, only AGREE analytical/metric approach uses all 12 principles of the GAC for the greenness assessment [42]. Hence, AGREE metric approach was applied for the greenness evaluation of the present sustainable HPTLC technique [42].

Based on all these assumptions and reports, the present study was an attempt to establish and validate a rapid, highly sensitive and green/sustainable HPTLC technique for the simultaneous estimation of CCHO and EOH in essential oils and traditional and ultrasound-assisted methanolic extracts of three different species of cinnamon including *C. zeylanicum, C. burmannii*, and *C. cassia*. The present sustainable HPTLC technique for the simultaneous estimation of CCHO and EOH was validated in terms of “linearity, system suitability parameters, accuracy, precision, robustness, sensitivity, and specificity/peak purity” according to The International Council for Harmonization for the Technical Requirements for Pharmaceuticals for Human Use (ICH) Q2 (R1) guidelines [45]. 

## 2. Results and Discussion

### 2.1. Method Development

**Figure 1:**
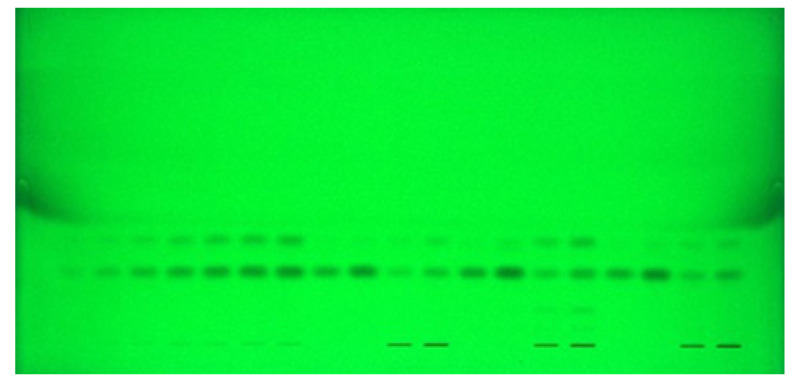
A representative pictogram for the developed high-performance thin-layer chromatography (HPTLC) plate for the simultaneous estimation of cinnamaldehyde (CCHO) and eugenol (EOH).

For the simultaneous estimation of CCHO and EOH, different proportions of cyclohexane and ethyl acetate such as cyclohexane/ethyl acetate (50:50, *v v*^−1^), cyclohexane/ethyl acetate (60:40, *v v*^−1^), cyclohexane/ethyl acetate (70:30, *v v*^−1^), cyclohexane/ethyl acetate (80:20, *v v*^−1^), and cyclohexane/ethyl acetate (90:10, *v v*^−1^) were evaluated as the solvent systems for the development of a suitable band. All investigated solvent systems were developed under chamber saturation conditions (Figure 1).

**Figure 2:**
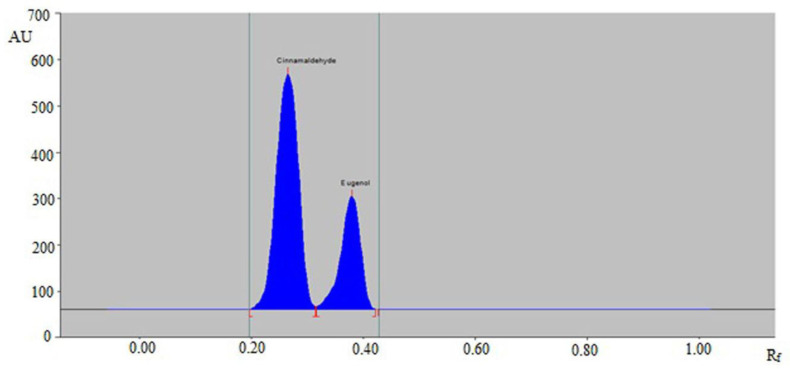
Representative chromatograms of standard CCHO and EOH.

From the obtained results, it was observed that the solvent systems cyclohexane/ethyl acetate (50:50, *v v*^−1^), cyclohexane/ethyl acetate (60:40, *v v*^−1^), cyclohexane/ethyl acetate (70:30, *v v*^−1^), and cyclohexane/ethyl acetate (80:20, *v v*^−1^) offered the poor densitometry peaks of CCHO and EOH with poor asymmetry/tailing factor (As) values for CCHO (As ˃ 1.30) and EOH (As ˃ 1.35). However, when the solvent system cyclohexane/ethyl acetate (90:10, *v v*^−1^) was studied, it was observed that this solvent system offered a well-separated and intact chromatographic peak of CCHO at R_f_ = 0.27 ± 0.01 and of EOH at R_f_ = 0.38 ± 0.01 (Figure 2). In addition, the As values of CCHO and EOH were found to be 1.04 and 1.10, which are highly acceptable. Accordingly, the cyclohexane/ethyl acetate (90:10, *v v*^−1^) solvent system was optimized as the mobile phase for the simultaneous estimation of CCHO and EOH in essential oils and traditional and ultrasound-assisted extracts of different species of cinnamon. The spectral bands for CCHO and EOH were obtained under densitometry mode and maximum response under reflectance/absorbance mode was obtained at the wavelength (λ_max_) = 296 nm for CCHO and EOH (Supplementary Materials Table S1). Hence, the entire simultaneous estimation of CCHO and EOH was carried out at λ_max_ = 296 nm.

### 2.2. Method Validation

Different validation parameters for the simultaneous estimation of CCHO and EOH were determined as per ICH-Q2 (R1) guidelines [45]. The results for the least square regression analysis of calibration curves (CCs) of CCHO and EOH are included in Table 1.

The calibration curve (CC) for CCHO and EOH was observed as linear in the range of 10–2000 ng band^−1^. The results showed a good linear relationship between the concentration and spot area of CCHO and EOH. The determination coefficient (R^2^) value for CCHO and EOH was obtained as 0.9986 and 0.9991, respectively, which were highly significant (*p* < 0.05). All these observations and results suggested that the sustainable HPTLC technique was linear and acceptable for the simultaneous estimation of CCHO and EOH.

The system suitability parameters for the sustainable HPTLC technique were determined and results tabulated in Table 2. The retention factor (R_f_) value, As value and several theoretical plates per meter (N m^−1^) for the sustainable HPTLC technique were found to be acceptable for the simultaneous estimation of CCHO and EOH.

The accuracy of the sustainable HPTLC technique for the simultaneous estimation of CCHO and EOH was estimated as % recovery and results are included in Table 3.

The % recoveries of CCHO and EOH at three different quality control (QC) levels were estimated as 98.45–101.16 and 99.32–100.84%, respectively using the sustainable HPTLC technique. The estimated % recoveries within the limit of 100 ± 2% for CCHO and EOH showed that the sustainable HPTLC technique was accurate for the simultaneous estimation of CCHO and EOH. 

The precision of the sustainable HPTLC technique for the simultaneous estimation of CCHO and EOH was determined in terms of instrumental and intra/inter-assay precision and expressed as the percent of the coefficient of variation (% CV). The results for instrumental precision are included in Table S2. The % CVs for CCHO and EOH were estimated as 0.63 and 0.75%, respectively. The results of intra/inter-assay precisions for the simultaneous estimation of CCHO and EOH using the sustainable HPTLC technique are included in Table 4.

The % CVs of CCHO and EOH for the intra-assay precision were predicted as 0.52–0.73 and 0.48–0.65 %, respectively. The % CVs of CCHO and EOH for inter-assay precision were predicted as 0.55–0.88 and 0.55–0.75%, respectively. The predicted values of % CV of CCHO and EOH for instrumental and intra/inter-assay precisions within ± 2% magnitude indicated that the sustainable HPTLC technique was precise enough for the simultaneous estimation of CCHO and EOH.

The robustness of the sustainable HPTLC technique for the simultaneous estimation of CCHO and EOH was evaluated by introducing small deliberate changes in the composition of mobile phase components, total run length, saturation time, and detection wavelength. The results of robustness analysis after changing mobile phase components are included in Table 5. The % CVs for CCHO and EOH after this change were determined as 0.60–0.68 and 0.75–0.85%, respectively. In addition, the R_f_ values for CCHO and EOH were recorded as 0.26–0.28 and 0.37–0.39, respectively.

The results of robustness analysis after changing total run length are included in Table S3. The % CVs for CCHO and EOH after this change were determined as 0.59–0.68 and 0.67–0.90%, respectively. In addition, the R_f_ values for CCHO and EOH were recorded as 0.25–0.29 and 0.36–0.40, respectively. The results of robustness analysis after changing saturation time are included in Table S4. The % CVs for CCHO and EOH after this change were determined as 0.66–0.71 and 0.58–0.85%, respectively. In addition, the R_f_ values for CCHO and EOH were recorded as 0.26–0.27 and 0.37–0.38, respectively. The results of robustness analysis after changing detection wavelength are included in Table S5. The % CVs for CCHO and EOH after this change were determined as 0.59–0.71 and 0.61–0.69%, respectively. In addition, the R_f_ values for CCHO and EOH were recorded as 0.27 and 0.38, respectively. The small variations in R_f_ values and lower % CVs after changing different chromatographic conditions showed that the sustainable HPTLC technique was robust for the simultaneous estimation of CCHO and EOH.

The sensitivity of the sustainable HPTLC technique for the simultaneous estimation of CCHO and EOH was determined in terms of detection (LOD) and quantification (LOQ) limits and results are presented in Table 1. The LOD and LOQ values for CCHO were determined as 3.56 *±* 0.08 and 10.68 *±* 0.24 ng band^−1^, respectively. However, the LOD and LOQ values for EOH were determined as 3.62 ± 0.09 and 10.86 ± 0.27 ng band^−1^, respectively. The recorded values of LOD and LOQ suggested that the sustainable HPTLC technique was highly sensitive for the simultaneous detection and quantification of CCHO and EOH. 

**Figure 3:**
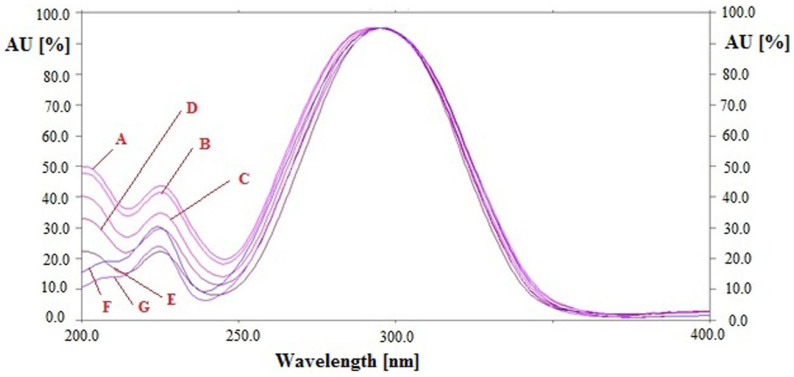
Overlaid ultra-violet (UV) absorption spectra of (A) standard CCHO and EOH, (B) CCHO and EOH in essential oil of *Cinnamomum zeylanicum*, (C) CCHO and EOH in essential oil of *C. burmannii*, (D) CCHO and EOH in essential oil of *C. cassia*, (E) CCHO and EOH in methanolic extract of *C. zeylanicum*, (F) CCHO and EOH in methanolic extract of *C. burmannii* and (G) CCHO and EOH in methanolic extract of *C. cassia*.

The specificity/peak purity of the sustainable HPTLC technique for the simultaneous estimation of CCHO and EOH was determined by comparing the R_f_ values and overlaid UV-absorption spectra of CCHO and EOH in essential oils and traditional and ultrasound-assisted methanolic extract of different species of cinnamon (*C. zeylanicum, C. burmannii*, and *C. cassia*) with that of standards CCHO and EOH. The overlaid UV spectra of standards CCHO and EOH and CCHO and EOH in essential oils and traditional and ultrasound-assisted methanolic extract of different species of cinnamon (*C. zeylanicum, C. burmannii*, and *C. cassia*) are summarized in Figure 3.

The maximum chromatographic response of CCHO and EOH in standards and essential oils and traditional and ultrasound-assisted methanolic extract of different species of cinnamon (*C. zeylanicum, C. burmannii*, and *C. cassia*) were recorded at λ_max_ = 296 nm under reflectance/absorbance mode. The similar UV-absorption spectra, R_f_ values and λ_max_ of CCHO and EOH in standards and essential oils and traditional and ultrasound-assisted methanolic extract of different species of cinnamon (*C. zeylanicum, C. burmannii*, and *C. cassia*) indicated that the sustainable HPTLC technique was specific for the simultaneous estimation of CCHO and EOH.

### 2.3. Application of Sustainable HPTLC Technique in the Simultaneous Estimation of CCHO and EOH in Essential Oils, Traditional and Ultrasound-Assisted Extracts of Different Species of Cinnamon

**Figure 4:**
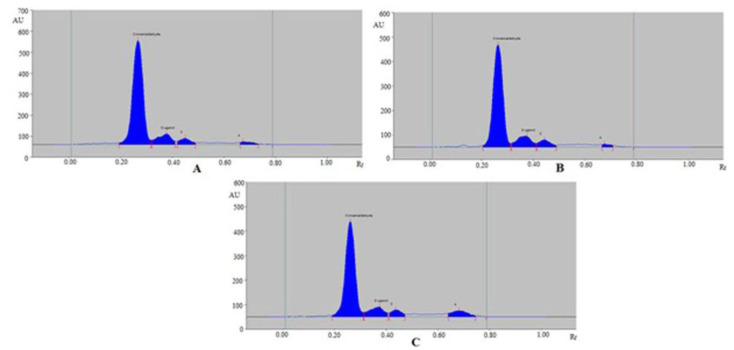
Representative chromatograms of CCHO and EOH in (**A**) essential oil of *C. zeylanicum*, (**B**) essential oil of *C. burmannii* and (**C**) essential oil of *C. cassia*.

**Figure 5:**
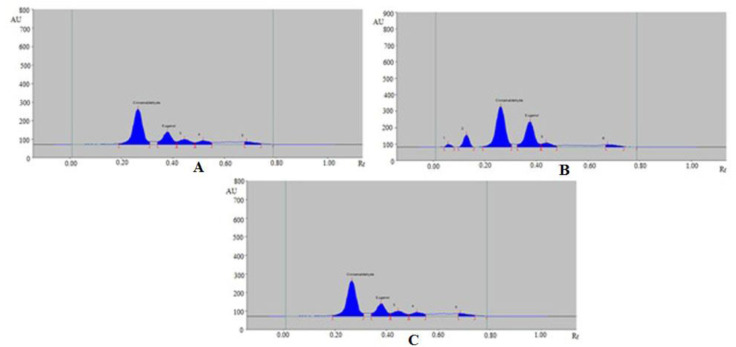
Representative chromatograms of CCHO and EOH in (**A**) methanolic extract of *C. zeylanicum*, (**B**) methanolic extract of *C. burmannii* and (**C**) methanolic extract of *C. cassia*.

The sustainable HPTLC technique could be an alternative approach to conventional analytical techniques for the simultaneous estimation of CCHO and EOH in essential oils and traditional and ultrasound-assisted methanolic extract of different species of cinnamon (*C. zeylanicum*, *C. burmannii*, and *C. cassia*). The chromatograms of CCHO and EOH from essential oils and traditional and ultrasound-assisted methanolic extract of different species of cinnamon (*C. zeylanicum, C. burmannii*, and *C. cassia*) were verified by comparing their TLC spot at R_f_ = 0.27 ± 0.01 for CCHO and R_f_ = 0.38 ± 0.01 for EOH with that of standards CCHO and EOH. The representative chromatograms of CCHO and EOH in essential oils of different species of cinnamon are presented in Figure 4, which presented similar peaks of CCHO and EOH to that of standards CCHO and EOH in all three species of cinnamon. In addition, 2 additional peaks were also found in essential oils of all three different species of cinnamon. The representative chromatograms of CCHO and EOH in traditional methanolic extracts of different species of cinnamon are presented in Figure 5 which also presented similar peaks of CCHO and EOH to that of standards CCHO and EOH in all three species of cinnamon. In addition, 3, 4, and 3 additional peaks were also recorded in *C. zeylanicum*, *C. burmannii*, and *C. cassia*, respectively.

**Figure 6:**
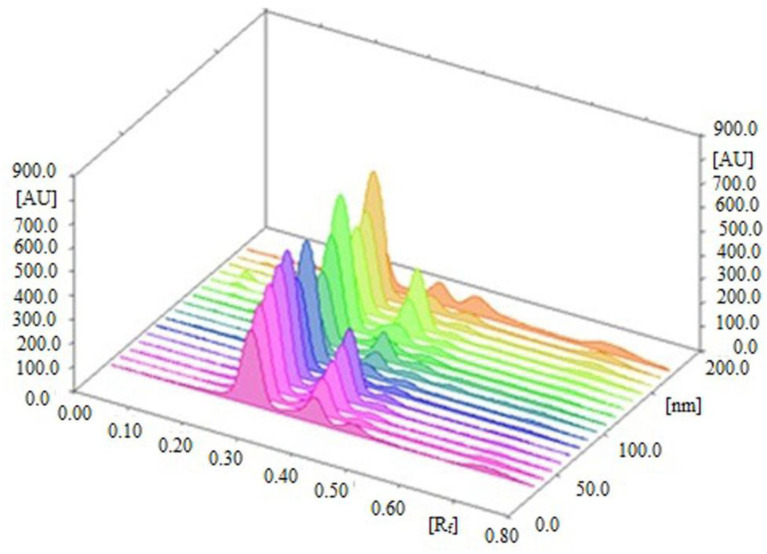
3D view of CCHO, EOH, essential oils, and methanolic extracts.

The 3D track spectra of CCHO and EOH in standards and all studied samples of cinnamon species are presented in Figure 6. The presence of additional peaks in all studied samples of cinnamon suggested that the sustainable HPTLC technique can be successfully applied for the simultaneous estimation of CCHO and EOH in the presence of other phytoconstituents/impurities. The contents of CCHO and EOH in essential oils and traditional and ultrasound-assisted methanolic extract of different species of cinnamon (*C. zeylanicum*, *C. burmannii*, and *C. cassia*) were determined from the CCs of CCHO and EOH and results are included in Table 6.

The contents of CCHO in essential oils of *C. zeylanicum, C. burmannii*, and *C. cassia* were determined as 191.20 ± 3.95, 214.24 ± 4.34, and 202.09 ± 4.17 mg g^−1^, respectively. The contents of CCHO in traditional methanolic extracts of *C. zeylanicum, C. burmannii*, and *C. cassia* were determined as 96.36 ± 2.79, 118.49 ± 2.97, and 114.18 ± 2.84 mg g^−1^, respectively. However, the contents of CCHO in ultrasound-assisted methanolic extracts of *C. zeylanicum, C. burmannii*, and *C. cassia* were determined as 111.57 ± 3.11, 134.39 ± 3.28, and 129.07 ± 3.04 mg g^−1^, respectively. The contents of EOH in essential oils of *C. zeylanicum, C. burmannii*, and *C. cassia* were determined as 61.26 ± 1.78, 79.21 ± 1.89, and 69.02 ± 1.91 mg g^−1^, respectively. The contents of EOH in traditional methanolic extracts of *C. zeylanicum*, *C. burmannii*, and *C. cassia* were determined as 73.38 ± 1.95, 165.41 ± 2.41, and 109.10 ± 1.38 mg g^−1^, respectively. However, the contents of EOH in ultrasound-assisted methanolic extracts of *C. zeylanicum, C. burmannii*, and *C. cassia* were determined as 87.20 ± 2.04, 218.09 ± 2.88, and 121.85 ± 1.57 mg g^−1^, respectively. The contents of CCHO were found to be significantly higher in essential oils of all three different species of cinnamon compared to their traditional and ultrasound-assisted methanolic extracts (*p* < 0.05). However, the contents of EOH were significantly higher in traditional and ultrasound-assisted methanolic extracts of all three different species of cinnamon compared to their essential oils (*p* < 0.05). These observations indicated that the CCHO is present in higher amounts in essential oils of different cinnamon species, while the EOH is present in higher amounts in methanolic extracts of different species of cinnamon. The contents of CCHO and EOH in ultrasound-assisted methanolic extracts of *C. zeylanicum, C. burmannii*, and *C. cassia* were significantly higher compared to their traditional methanolic extracts (*p* < 0.05). Based on all these observations and results, the ultrasound method for the extraction of CCHO and EOH has been considered superior to its traditional method of extraction. Overall, these results indicated that the sustainable HPTLC technique can be successfully applied in the simultaneous estimation of CCHO and EOH in the wide variety of plants and herbal products containing CCHO and EOH as the main phytoconstituents.

### 2.4. Greenness Evaluation Using AGREE

**Figure 7:**
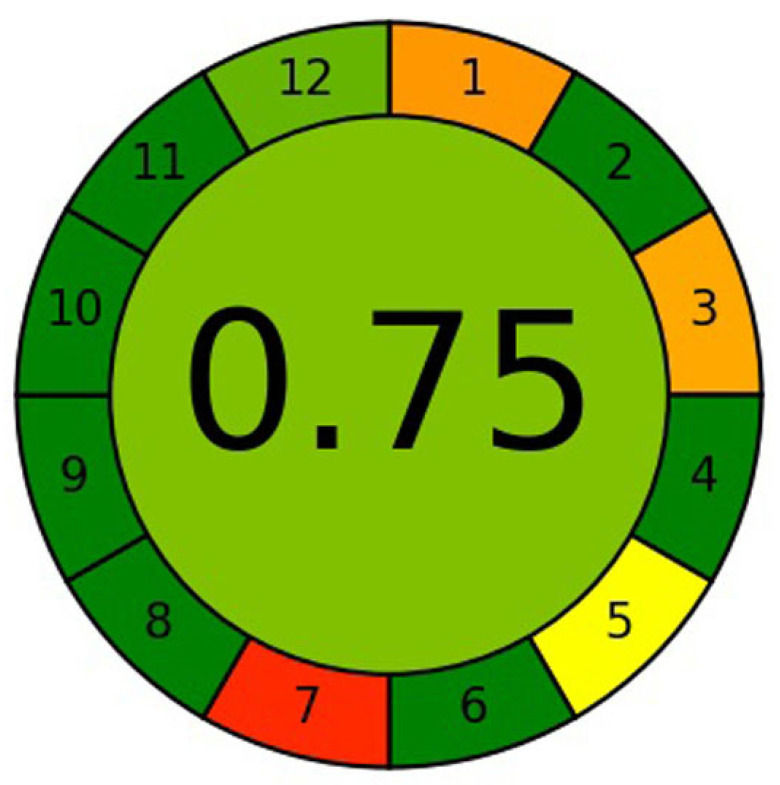
The representative pictogram for AGREE analytical score for sustainable HPTLC technique obtained using AGREE: The Analytical Greenness Calculator.

Different analytical/metric approaches have been applied for the evaluation of greenness profiles of analytical techniques [40,41,42,43,44]. Among them, only AGREE uses all 12 principles GAC for this purpose [42]. Hence, the greenness profile of the sustainable HPTLC technique was obtained using AGREE: The Analytical Greenness Calculator (version 0.5, Gdansk University of Technology, Gdansk, Poland, 2020) in this work. The representative pictogram for the AGREE score of the sustainable HPTLC technique is presented in Figure 7. The AGREE score of the sustainable HPTLC technique was estimated as 0.75, suggested the excellent greenness of the present HPTLC technique for the simultaneous estimation of CCHO and EOH in essential oils and traditional and ultrasound-assisted methanolic extracts of different species of cinnamon.

### 2.5. Literature Comparison

The sustainable HPTLC technique for the simultaneous estimation of CCHO and EOH was compared with different analytical techniques reported for the simultaneous estimation of CCHO and EOH. The results of different validation parameters of present HPTLC technique compared to literature analytical techniques are included in Table 7.

Three different validation parameters such as linearity range, accuracy, and precision of the sustainable HPTLC technique were compared with reported analytical techniques. The linearity range, accuracy, and precision (as % CV) of the literature GC-FID technique for the simultaneous estimation of CCHO and EOH were recorded as 0.45–452 µg mL^−1^, 84–111%, and 4.9–5.4%, respectively for CHO and 0.31–625 µg mL^−1^, 88–96%, and 4.5–8.7%, respectively for EOH [30]. These validation parameters of the literature GC-FID technique were much inferior to the sustainable HPTLC technique. Similarly, the linearity range, accuracy, and precision of one of the reported HPLC techniques for the simultaneous estimation of CCHO and EOH were inferior to the sustainable HPTLC technique [31]. The linearity range of another HPLC technique for the simultaneous estimation of CCHO and EOH was also inferior to the sustainable HPTLC technique, while the accuracy and precision of this technique were within the limit of ICH guidelines [32]. The accuracy and precisions of literature HPTLC technique for the simultaneous estimation of CCHO and EOH were also found to be acceptable and within the limit of ICH guidelines. However, its linearity range was much inferior to the sustainable HPTLC technique [33]. Based on all these observations and comparisons, the present sustainable HPTLC technique was found to be suitable and highly sensitive for the simultaneous estimation of CCHO and EOH.

## 3. Materials and Methods

### 3.1. Materials

The working standards of CCHO and EOH were procured from Sigma Aldrich (St. Louis, MO, USA). HPLC grades cyclohexane, ethyl acetate and methanol were obtained from E-Merck (Darmstadt, Germany). The barks of different species of cinnamon (*C. zeylanicum, C. burmannii*, and *C. cassia*) were obtained randomly from the hypermarket in Al-Kharj, Saudi Arabia. The essential oils and extracts of different species of cinnamon (*C. zeylanicum, C. burmannii*, and *C. cassia*) were obtained in the laboratory. All the reagent/solvents were of analytical/pharmaceutical grades.

### 3.2. Instrumentation and Analytical Conditions

The simultaneous estimation of CCHO and EOH was performed using the instrumentations and analytical conditions summarized in Table S1.

### 3.3. CC of CCHO and EOH

The standard solution (SS) of CCHO and EOH was obtained separately by dissolving the required amounts of both compounds in the required quantity of methanol in such a way that the final SS of 100 μg mL^−1^ for both compounds was achieved. Serial dilutions of SS of CCHO and EOH were then prepared by taking variable volumes of CCHO SS or EOH SS and diluting using methanol to obtain the concentrations in the range 10–2000 ng band^−1^ for CCHO and EOH. Around 200 μL of each concentration of CCHO and EOH was applied on TLC plates and the spot area of each concentration was noted. The CC for CCHO and EOH was prepared by plotting the concentrations on the x-axis and spot area on the y-axis in six replicates (n = 6).

### 3.4. Extraction Procedure for Different Species of Cinnamon

Accurately weighed 10 g of the dried cinnamon barks of different species (*C. zeylanicum*, *C. burmannii*, and *C. cassia*) were refluxed with methanol (100 mL) for 1 h in a water bath and filtered through Whatman filter paper (No. 41). The marc left out was refluxed again three times with 70 mL of methanol for 1 h and filtered again. The methanol was evaporated using a rotary vacuum evaporator, and the residue was dissolved in 250 mL of methanol in a volumetric flask. The procedure was repeated for three times (n = 3). These solutions were used as the test solutions for the simultaneous estimation of CCHO and EOH in the traditional methanolic extracts of three different species of cinnamon bark using the sustainable HPTLC technique.

### 3.5. Ultrasound-Assisted Extraction Procedure for Different Species of Cinnamon

The ultrasound-assisted extraction of the dried cinnamon barks of different species (*C. zeylanicum*, *C. burmannii*, and *C. cassia*) was performed using ultrasonic vibrations using the Bransonic series (Model CPX5800H-E; Princeton, NJ, USA). A total of 10 g of dried cinnamon bark of different species was taken and extracted with 100 mL of methanol. The methanol was evaporated using a rotary vacuum evaporator and the residue was dissolved in 50 mL of methanol in a volumetric flask. It was ultrasonicated at 50 °C for 1 h. This procedure was repeated three times (n = 3). These solutions were used as the test solutions for the simultaneous estimation of CCHO and EOH in the ultrasound-assisted methanolic extracts of cinnamon bark using the sustainable HPTLC technique.

### 3.6. Isolation of Essential Oils from Different Species of Cinnamon

The essential oils of different species of cinnamon barks (*C. zeylanicum*, *C. burmannii*, and *C. cassia*) were obtained by a hydro-distillation method according to the standard method of Egyptian Pharmacopoeia. Around 150 g of dried cinnamon barks of different species of cinnamon (*C. zeylanicum*, *C. burmannii*, and *C. cassia*) were used for essential oil extraction under a Clevenger trap apparatus. The specified amount of cinnamon bark was mixed with 1000 mL of water for 8 h distillations. The oil layer and water separation were trapped with ethyl acetate (3 × 50 mL). Furthermore, the organic layer was concentrated under a rotary vacuum evaporator to obtain the pure essential oil for each species of cinnamon.

### 3.7. Method Validation

The present sustainable HPTLC technique for the simultaneous estimation of CCHO and EOH was validated in terms of “linearity, system suitability parameters, precision, accuracy, robustness, sensitivity, and peak purity/specificity” as per ICH-Q2 (R1) guidelines [45]. The linearity range of CCHO and EOH was evaluated by plotting the concentrations of CCHO and EOH against their spot area. The linearity for CCHO and EOH was evaluated in the range of 10–2000 ng band^−1^ (n = 6). The accuracy of the sustainable HPTLC technique for CCHO and EOH was evaluated as % recovery. The accuracy was determined at three different QC samples including lower quality control (LQC; 50 ng band^−1^), middle quality control (MQC; 500 ng band^−1^), and high-quality control (HQC; 1000 ng band^−1^) samples for CCHO and EOH. The % recovery was estimated at each QC level of CCHO and EOH (n = 6).

The system suitability parameters for the sustainable HPTLC technique for the simultaneous estimation of CCHO and EOH were obtained by the determination of R_f_, As, and N m^−1^. The values of R_f_, As, and N m^−1^ were obtained using their standard formulae, reported in our latest publication [43].

The precision of sustainable HPTLC technique for CCHO and EOH was evaluated in terms of instrumental and intra/inter-assay precision. The instrumental precision was evaluated by the repeatable injections of same spectral band many times for the same solution of fixed concentration (n = 6). The instrumental precision was determined at MQC (500 ng band^−1^) for CCHO and EOH. Intra-assay precision for CCHO and EOH was determined by the analysis of freshly prepared CCHO and EOH solutions at LQC, MQC, and HQC on the same day for the sustainable HPTLC technique (n = 6). The inter-assay precision for CCHO and EOH was determined by the analysis of freshly prepared solutions at LQC, MQC, and HQC on three different days for the sustainable HPTLC technique (n = 6).

The robustness for CCHO and EOH was evaluated by introducing small deliberate changes in the chromatographic conditions which includes minor modifications in mobile phase composition, total run length, saturation time, and detection wavelength for the sustainable HPTLC technique. The original mobile phase system cyclohexane/ethyl acetate (90:10, *v v*^−1^) was modified into cyclohexane/ethyl acetate (92:8, *v v*^−1^) and cyclohexane/ethyl acetate (88:12, *v v*^−1^) for CCHO and EOH and the chromatographic response was noted (n = 6). The total run length of CCHO and EOH was changed from 80 mm to 82 mm and 78 mm for the sustainable HPTLC technique and the chromatographic response was recorded. The saturation time of CCHO and EOH was changed from 30 min to 32 min and 28 min and the chromatographic response was recorded for the sustainable HPTLC technique [25]. The detection wavelength for CCHO and EOH was changed from 296 nm to 298 nm and 294 nm and the chromatographic response was recorded for the sustainable HPTLC technique [43].

The sensitivity of the sustainable HPTLC technique for *CC*HO and EOH was determined in terms of LOD and LOQ using standard deviation method. The LOD and LOQ values for CCHO and EOH were determined using their standard formulae, reported previously (n = 6) [45].

The specificity/peak purity of the sustainable HPTLC technique for CCHO and EOH was determined by comparing the R_f_ values and UV-absorption spectra of CCHO and EOH in the traditional and ultrasound-assisted methanolic extract of *C. zeylanicum, C. burmannii* and *C. cassia* and essential oils of *C. zeylanicum, C. burmannii* and *C. cassia* with that of standard CCHO and EOH.

### 3.8. Application of Sustainable HPTLC Technique in the Simultaneous Estimation of CCHO and EOH in Essential Oils, Traditional and Ultrasound-Assisted Extracts of Different Species of Cinnamon

The prepared samples of essential oils, traditional, and ultrasound-assisted extracts of *C. zeylanicum, C. burmannii* and *C. cassia* were spotted on TLC plates and their chromatographic responses were recorded under the same experimental conditions and procedures used for the simultaneous estimation of standard CCHO and EOH (n = 3). The contents of CCHO and EOH in all studied samples were calculated using the CC of CCHO and EOH.

### 3.9. Greenness Evaluation Using AGREE

The greenness score for the sustainable HPTLC technique for the simultaneous estimation of CCHO and EOH was assessed using all 12 principles of GAC, described in the literature [42]. The AGREE scores (0.0–1.0) for the sustainable HPTLC technique was predicted using AGREE: The Analytical Greenness Calculator (version 0.5, Gdansk University of Technology, Gdansk, Poland, 2020) for the sustainable HPTLC technique.

### 3.10. Statistical Evaluation

All the values are presented as mean ± SD of three or six replicates. The statistical evaluation was performed by applying Dunnett’s test using GraphPad Prism software (version 6, GraphPad, San Diego, CA, USA). The *p*-value of less than 0.05 was considered to be statistically significant value.

## 4. Conclusions

The sustainable/green analytical techniques for the simultaneous estimation of CCHO and EOH are scarce in the literature. Hence, the present work was an attempt to establish and validate the rapid, highly sensitive, and sustainable HPTLC technique for the simultaneous estimation of CCHO and EOH in essential oils and traditional and ultrasound-assisted methanolic extracts of three different species of cinnamon. The sustainable HPTLC technique is simple, rapid, accurate, precise, robust, highly sensitive, sustainable, and specific for the simultaneous estimation of CCHO and EOH. The contents of CCHO and EOH were found to be higher in ultrasound-assisted methanolic extracts of all three different species of cinnamon compared to its traditional methanolic extracts. Hence, the ultrasound-based extraction of CCHO and EOH from different species of cinnamon has been considered superior to the traditional method of extraction. The AGREE score indicated the excellent greenness profile of the sustainable HPTLC technique. The present sustainable technique has been found to be superior to literature analytical techniques for the simultaneous estimation of CCHO and EOH. Based on all these observations and results, the proposed sustainable HPTLC technique can be successfully applied in the simultaneous estimation of CCHO and EOH in the wide variety of plant extracts and herbal products containing CCHO and EOH as the active phytoconstituents.

## Figures and Tables

**Table 1:** Results for least square regression analysis for the simultaneous estimation of cinnamaldehyde (CCHO) and eugenol (EOH) using sustainable/green high-performance thin-layer chromatography (HPTLC) technique (mean ± SD; n = 6).

Parameters	CCHO	EOH
Linearity range (ng band^−1^)	10–2000	10–2000
R^2^	0.9986	0.9991
Slope ± SD	35.00 ± 1.58	16.11 ± 1.05
Intercept ± SD	1586.60 ± 16.32	446.61 ± 4.67
Standard error of slope	0.64	0.42
Standard error of intercept	6.66	1.90
95% confidence interval of slope	32.22–37.77	14.27–17.96
95% confidence interval of intercept	1557.52–1615.27	438.40–454.81
LOD ± SD *(*ng band^−1^*)*	3.56 ± 0.08	3.62 ± 0.09
LOQ ± SD *(*ng band^−1^*)*	10.68 ± 0.24	10.86 ± 0.27

**Table 2:** System suitability parameters in terms of retention factor (R_f_), asymmetry/tailing factor (As) and several theoretical plates per meter (N m^−1^) of CCHO and EOH for sustainable HPTLC technique.

Parameters	CCHO	EOH
R_f_	0.27	0.38
As	1.04	1.10
N m^−1^	5248	4146

**Table 3:** Measurement of the accuracy of CCHO and EOH for sustainable HPTLC technique (mean ± SD; n = 6).

Conc. *(*ng band^−1^*)*	Conc. Found (ng band^−1^) ± SD	Recovery (%)	CV (%)
	**CCHO**		
50	50.58 ± 0.42	101.16	0.80
500	492.26 ± 2.67	98.45	0.54
1000	989.34 ± 5.08	98.93	0.51
	**EOH**		
50	49.68 ± 0.35	99.36	0.70
500	504.21 ± 2.85	100.84	0.56
1000	993.24 ± 5.53	99.32	0.55

**Table 4:** Measurement of intra/interday precision of CCHO and EOH for sustainable HPTLC technique (mean ± SD; n = 6).

Conc. (ng band^−1^)	Intraday Precision			Interday Precision		
	Conc. (ng band^−1^) ± SD	Standard Error	CV (%)	Conc. (ng band^−1^) ± SD	Standard Error	CV (%)
CCHO						
50	50.49 ± 0.37	0.15	0.73	49.23 ± 0.43	0.17	0.88
500	494.19 ± 2.89	1.18	0.58	504.32 ± 3.12	1.27	0.61
1000	992.56 ± 5.22	2.13	0.52	986.51 ± 5.44	2.22	0.55
EOH						
50	49.43 ± 0.32	0.32	0.65	49.28 ± 0.37	0.15	0.75
500	504.87 ± 2.90	2.90	0.57	495.89 ± 3.23	1.31	0.65
1000	995.51 ± 4.87	4.87	0.48	985.87 ± 5.50	2.42	0.55

**Table 5:** Results of robustness analysis of CCHO and EOH by changing mobile phase compositions for sustainable HPTLC technique (mean ± SD; n = 6).

Conc. (ng band^−1^)	Mobile Phase Composition (Cyclohexane/Ethyl Acetate)			Results		
	Original	Used		(ng band^−1^) ± SD	% CV	R_f_
**CCHO**						
		92:8	+2.0	511.23 ± 3.10	0.60	0.26
500	90:10	90:10	0.0	514.62 ± 3.35	0.65	0.27
		88:12	−2.0	516.41 ± 3.52	0.68	0.28
**EOH**						
		92:8	+2.0	489.22 ± 3.70	0.75	0.37
500	90:10	90:10	0.0	491.80 ± 3.90	0.79	0.38
		88:12	−2.0	498.28 ± 4.28	0.85	0.39

**Table 6:** Application of sustainable HPTLC method in simultaneous estimation of CCHO and EOH in methanolic extracts of C. zeylanicum, C. burmannii and C. cassia and essential oils of C. zeylanicum, C. burmannii and C. cassia produced by traditional and ultrasonication methods (mean *±* SD; n = 3).

Samples	Traditional Extraction	Ultrasonication-Based Extraction
**Amount of CCHO (mg g^−1^)**		
*C. zeylanicum* oil	191.20 ± 3.95	-
*C. burmannii* oil	214.24 ± 4.34	-
*C. cassia* oil	202.09 ± 4.17	-
*C. zeylanicum* extract	96.36 ± 2.79	111.57 ± 3.11
*C. burmannii* extract	118.49 ± 2.97	134.39 ± 3.28
*C. cassia* extract	114.18 ± 2.84	129.07 ± 3.04
**Amount of EOH (mg g^−1^)**		
*C. zeylanicum* oil	61.26 ± 1.78	-
*C. burmannii* oil	79.21 ± 1.89	-
*C. cassia* oil	69.02 ± 1.91	-
*C. zeylanicum* extract	73.38 ± 1.95	87.20 ± 2.04
*C. burmannii* extract	165.41 ± 2.41	218.09 ± 2.88
*C. cassia* extract	109.10 ± 1.38	121.85 ± 1.57

**Table 7:** Comparison of present sustainable/green HPTLC technique with literature analytical techniques for the simultaneous determination of CCHO and EOH.

Analytical Method	Compound						Ref.
	CCHO			EOH			
	Linearity Range	Accuracy (% Recovery)	Precision (% CV)	Linearity Range	Accuracy (% Recovery)	Precision (% CV)	
GC-FID	0.45–452 (µg mL^−1^)	84–111	4.9–5.4	0.31–625 (µg mL^−1^)	88–96	4.5–8.7	[30]
HPLC	1–200 (µg mL^−1^)	99.09	1.35–1.63	0.3–12 (µg mL^−1^)	99.20	1.43–1.61	[31]
HPLC	0.04–6.55 (µg mL^−1^)	95.95–99.86	0.35–2.89	0.45–36.00 (µg mL^−1^)	99.47–101.85	0.51–1.53	[32]
HPTLC	52.54–735.56 (ng band^−1^)	98.44–99.35	0.66–0.64	533.2–8531.2 (ng band^−1^)	98.25–99.32	0.34–1.09	[33]
HPTLC	10–2000 (ng band^−1^)	98.45–101.16	0.52–0.88	10–2000 (ng band^−1^)	99.32–100.84	0.48–0.75	Present work

## Data Availability

The data presented in this study are available in Supplementary Material.

## Supplementary Materials

The following are available online, Table S1: Chromatographic conditions and instrumentations used for the simultaneous estimation of CCHO and EOH for the sustainable HPTLC techniques, Table S2: Results of instrumental precision for the simultaneous estimation of CCHO and EOH for sustainable HPTLC technique (mean ± SD; n = 6), Table S3: Results of robustness analysis by changing total run length for the simultaneous estimation of CCHO and EOH using the sustainable HPTLC technique (mean ± SD; n = 6), Table S4: Results of robustness analysis by changing the saturation time for the simultaneous estimation of CCHO and EOH using the sustainable HPTLC technique (mean ± SD; n = 6), Table S5: Results of robustness analysis by changing the detection wavelength for simultaneous estimation of CCHO and EOH using the sustainable HPTLC technique (mean ± SD; n = 6).

## Sample Availability

Samples of the compounds CCHO and EOH are available from the authors.
