# Supplementary material for: Simultaneous Estimation of Cinnamaldehyde and Eugenol in Essential Oils and Traditional and Ultrasound-Assisted Extracts of Different Species of Cinnamon Using a Sustainable/Green HPTLC Technique
Source: Molecules. 2021 Apr 3;26(7):2054. doi: 10.3390/molecules26072054 (PMC8038348; doi:10.3390/molecules26072054)
Supplement: Supplementary file 1 [file molecules-26-02054-s001.pdf]

Supplementary materials

Simultaneous Estimation of Cinnamaldehyde and Eugenol in Essential Oils and Traditional and Ultrasound-Assisted Extracts of Different Species of Cinnamon Using a Sustainable/Green HPTLC Technique

Ahmed I. Foudah[1], Faiyaz Shakeel[2], Mohammed H. Alqarni[1], Samir A. Ross[3,4], Mohammad A. Salkini[1] and Prawez Alam[1*]

1 Department of Pharmacognosy, College of Pharmacy, Prince Sattam Bin Abdulaziz University, Al-Kharj 11942, Saudi Arabia; a.foudah@psau.edu.sa (A.I.F.); m.alqarni@psau.edu.sa (M.H.A.); m.salkini@psau.edu.sa (M.A.S.)

2 Department of Pharmaceutics, College of Pharmacy, King Saud University, Riyadh 11451, Saudi Arabia; faiyazs@fastmail.fm (F.S.)

3 *National Center for Natural Products Research,* University of Mississippi, Oxford, MS 38677, USA; sroos@olemiss.edu (S.A.R.)

4 *Department of Biomolecular Sciences, School of Pharmacy,* University of Mississippi, Oxford, MS 38677, USA

* Correspondence: p.alam@psau.edu.sa (P.A.)

Table S1. Chromatographic conditions and instrumentations used for the simultaneous estimation of CCHO and EOH for the sustainable HPTLC techniques.

Chromatographic conditions/instrumentation	CCHO	EOH
Instrument	CAMAG TLC system (CAMAG, Muttenz, Switzerland)	CAMAG TLC system (CAMAG, Muttenz, Switzerland)
Software	WinCAT's (version 1.4.3.6336, CAMAG, Muttenz, Switzerland)	WinCAT's (version 1.4.3.6336, CAMAG, Muttenz, Switzerland)
Syringe for sample application	CAMAG microliter Syringe (Hamilton, Bonaduz, Switzerland)	CAMAG microliter Syringe (Hamilton, Bonaduz, Switzerland)
TLC plates/stationary phase	10 x 20 cm glass backed plates pre-coated with NP silica gel 60 F254S plates (E-Merck, Darmstadt, Germany)	10 x 20 cm glass backed plates pre-coated with NP silica gel 60 F254S plates (E-Merck, Darmstadt, Germany)
Gas for sample application	Nitrogen	Nitrogen
Development chamber	CAMAG automatic developing chamber 2 (ADC2) (CAMAG, Muttenz, Switzerland)	CAMAG automatic developing chamber 2 (ADC2) (CAMAG, Muttenz, Switzerland)

Chamber saturation time	30 min	30 min
TLC Scanner	CAMAG TLC scanner-III (CAMAG, Muttenz, Switzerland)	CAMAG TLC scanner-III (CAMAG, Muttenz, Switzerland)
Mobile phase	Cyclohexane/ethyl acetate (90:10, *v v*$^{-1}$)	Cyclohexane/ethyl acetate (90:10, *v v*$^{-1}$)
Development distance on plate	80 mm	80 mm
Development mode	Linear ascending mode	Linear ascending mode
Sample application rate	150 nL s^{-1}	150 nL s^{-1}
Densitometry of scanning mode	Absorbance/reflectance	Absorbance/reflectance
Scanning wavelength of FBN	296 nm	296 nm

Table S2. Results of instrumental precision for the simultaneous estimation of CCHO and EOH for sustainable HPTLC technique (mean ± SD; n = 6).

Conc. (ng band^{-1})	Area ± SD	Standard error	CV (%)
	CCHO		
500	20141 ± 127	51.85	0.63
	EOH		
500	8298 ± 63	25.72	0.75

Table S3. Results of robustness analysis by changing total run length for the simultaneous estimation of CCHO and EOH using the sustainable HPTLC technique (mean ± SD; n = 6).

Conc.	Total run length (mm)			Results		
(ng band-1)	Original	Used		Area ± SD	% CV	Rf
		OHCC				
500	80	82	+2.0	18224 ± 108	0.59	0.29
		80	0.0	18762 ± 118	0.62	0.27
		78	-2.0	19245 ± 132	0.68	0.25
		HOE				
		Total run length (mm)				
500	80	82	+2.0	8302 ± 56	0.67	0.40
		80	0.0	8366 ± 65	0.77	0.38
		78	-2.0	8416 ± 76	0.90	0.36

Table S4. Results of robustness analysis by changing the saturation time for the simultaneous estimation of CCHO and EOH using the sustainable HPTLC technique (mean ± SD; n = 6).

Conc. (ng band⁻¹)	Saturation time (min)			Results		
	Original	Used		Area ± SD	% CV	R_f
CCHO						
		32	+2.0	18164 ± 121	0.66	0.27
500	30	30	0.0	19114 ± 133	0.69	0.27
		28	-2.0	19871 ± 143	0.71	0.26
EOH						
		32	+2.0	8178 ± 48	0.58	0.38
500	30	30	0.0	8256 ± 59	0.71	0.38
		28	-2.0	8298 ± 71	0.85	0.37

Table S5. Results of robustness analysis by changing the detection wavelength for simultaneous estimation of CCHO and EOH using the sustainable HPTLC technique (mean ± SD; n = 6).

Conc. (ng band⁻¹)	Detection wavelength (nm)			Results		
	Original	Used		Area ± SD	% CV	R_f
CCHO						
		298	+2.0	18674 ± 134	0.71	0.27
500	296	296	0.0	20224 ± 142	0.70	0.27
		294	-2.0	19876 ± 118	0.59	0.27
EOH						
Detection wavelength (nm)						
		298	+2.0	8283 ± 51	0.61	0.38
500	296	296	0.0	8412 ± 54	0.64	0.38
		294	-2.0	8362 ± 58	0.69	0.38
